# A Promising Approach to Integrally Evaluate the Disease Outcome of Cerebral Ischemic Rats Based on Multiple-Biomarker Crosstalk

**DOI:** 10.1155/2017/9506527

**Published:** 2017-05-25

**Authors:** Guimei Ran, Yixuan Wang, Haochen Liu, Chunxiang Wei, Tao Zhu, Haidong Wang, Hua He, Xiaoquan Liu

**Affiliations:** Center of Drug Metabolism and Pharmacokinetics, China Pharmaceutical University, Nanjing 210009, China

## Abstract

**Purpose:**

The study was designed to evaluate the disease outcome based on multiple biomarkers related to cerebral ischemia.

**Methods:**

Rats were randomly divided into sham, permanent middle cerebral artery occlusion, and edaravone-treated groups. Cerebral ischemia was induced by permanent middle cerebral artery occlusion surgery in rats. To form a simplified crosstalk network, the related multiple biomarkers were chosen as S100*β*, HIF-1*α*, IL-1*β*, PGI_2_, TXA_2_, and GSH-Px. The levels or activities of these biomarkers in plasma were detected before and after ischemia. Concurrently, neurological deficit scores and cerebral infarct volumes were assessed. Based on a mathematic model, network balance maps and three integral disruption parameters (*k*, *φ*, and *u*) of the simplified crosstalk network were achieved.

**Results:**

The levels or activities of the related biomarkers and neurological deficit scores were significantly impacted by cerebral ischemia. The balance maps intuitively displayed the network disruption, and the integral disruption parameters quantitatively depicted the disruption state of the simplified network after cerebral ischemia. The integral disruption parameter *u* values correlated significantly with neurological deficit scores and infarct volumes.

**Conclusion:**

Our results indicate that the approach based on crosstalk network may provide a new promising way to integrally evaluate the outcome of cerebral ischemia.

## 1. Introduction

Cerebral ischemia (CI) is a devastating clinical condition with high death and disability in human and brings deep frustration and despair to patients. It is well known that CI triggers a complex cascade of metabolic alterations involved in multiple biological pathways such as oxidative stress [1, 2], inflammation [3], neuronal damage [4], and hypoxia-inducible factor [5]. The biomarkers connect directly or indirectly with each other to form a complex network in the diverse pathways. Regarding the relationships between a single biomarker or a single pathway and the disease outcome of CI, the related studies have been more prominent. However, the integrative evaluation for the outcome of CI based on the related multiple biomarkers has not caused enough attention. Therefore, the objective of this research is to integrally evaluate the outcome of CI according to the integrated information from the related multiple biomarkers in the diverse intertwining pathways.

A substantial body of evidence suggests that considerable reactive oxygen species (ROS) play vital roles in tissue injury in acute cerebral ischemic stroke [1, 2, 6]. Thereby, as a therapeutic target, ROS have been studied in recent years. ROS can be scavenged by endogenous antioxidant enzymes such as superoxide dismutase (SOD) and glutathione peroxidase (GSH-Px). Thus, the activity of GSH-Px is proved to reflect oxidative stress level. Various stimuli, such as ROS and CI [7, 8], are able to activate hypoxia-inducible factor 1 (HIF-1). HIF-1 is a master transcription factor of oxygen homeostasis consisting of an oxygen-regulated subunit (HIF-1*α*) and a constitutively expressed subunit (HIF-1*β*) [9, 10]. In most cases, the activity of HIF-1 depends on the availability and activity of HIF-1*α* subunit [11, 12]. HIF-1 performs its molecular function via binding to target genes and the following promotion of target gene transcription implicated with erythropoiesis (EPO) [10], vascular endothelial growth factor (VEGF) [13] and the receptor for advanced glycation end products (RAGE) [14]. RAGE, a multiple-ligand receptor of the immunoglobulin superfamily, recognizes multiple ligands including AGE, amyloid-*β*-peptide, amphoterin, *β*-fibrils, and S100*β* [15]. The elevated S100*β* levels are observed in cerebrospinal fluid (CSF) and peripheral blood under many pathological conditions, such as ischemic stroke and traumatic brain injury [16–18]. It is shown that S100*β*-mediated proinflammatory responses are RAGE-dependent in microglia. For example, S100*β* engagement of RAGE induces expression of the proinflammatory enzyme cyclooxygenase-2 (COX-2) [19]. Moreover, RAGE ligation by S100*β* synergizes with IL-1*β* and TNF-*α* to upregulate COX-2 expression [19]. In addition, S100*β* stimulates IL-1*β* production in astrocytes and microglia [20, 21]. Therefore, S100*β* may be used as a common reporter for brain damage after ischemic or traumatic brain injury onset. Thromboxane A_2_ (TXA_2_) and prostacyclin (PGI_2_) are downstream products of COX-2. TXA_2_ induces platelet aggregation, vasoconstriction, and endothelium injury, while PGI_2_, an endogenous antagonist of TXA_2_, possesses the opposite biological activities [22–24]. Maintaining homeostasis of PGI_2_/TXA_2_ ratio can play an important role in regulating cerebral blood flow to respond with ischemia [25]. In contrast, the imbalance in PGI_2_ and TXA_2_ production is involved in the pathophysiology of myocardial infarction, atherosclerosis, and CI [23, 26, 27]. In recent years, more attentions have been focused on the functions of PGI_2_ and TXA_2_ in regulating blood coagulation and vascular constriction [28, 29]. These studies suggest that the ratio of PGI_2_ and TXA_2_ may reflect platelet and endothelial function in CI. It is well known that an inflammatory response contributes to ischemic stroke outcomes [30]. At the present time, inflammatory cytokines including IL-1*β*, IL-6, and TNF-*α* have been confirmed to be related to ischemic stroke [31–33]. Moreover, IL-1*β* induces S100*β* secretion in cortical primary astrocyte cultures, C6 glioma cells, and hippocampal slices of rats [34]. The mutual induction of IL-1*β* and S100*β* supports that “cytokine cycle” participates in the genesis of CNS diseases [34]. Therefore, the concentration of IL-1*β* is used to reflect the inflammatory level in the CNS.

The related biomarkers form a complex crosstalk network in multiple metabolic pathways. The crosstalk network shows robustness through the interconnection and interaction of the multiple metabolic pathways. Owing to the robustness property of the crosstalk network, biological systems could prevent and counteract perturbations to maintain physiological homeostasis in a variety of physiological conditions [35, 36]. However, the physiological homeostasis is disrupted by diseases such as CI. Thus, it is unfeasible to assess the complex state of CI using a single biomarker or a single pathway. Meanwhile, it is also difficult to evaluate CI outcome using all the related biomarkers. Therefore, in this study, a simplified crosstalk network (mini network) is established according to the interactions among the related biomarkers. We hypothesized that the multiple biomarkers in the mini network, based on a mathematical model, could provide more valuable integrative information for evaluating the disease outcome.

CI triggers a complex cascade of metabolic alterations involved in multiple biological pathways. Edaravone (EDA), a free radical scavenger, has been widely used to treat cerebral infarction in Japan since 2001. It is reported that EDA has multitarget pharmacologies such as oxidant stress and inflammation [37–39]. Furthermore, we also wondered whether the improvement of the disease state correlated with the changes of the parameter values after MCAO. Therefore, we designed an EDA-treated group. To obtain the change information of these related biomarkers after MCAO, we designed an MCAO group. Meanwhile, to eliminate the impact of surgical injuries on the levels or activities of these biomarkers, we designed a SHAM group. In this work, we designed a mathematical model to integrate the information from these related biomarkers into three disruption parameters (*k*, *φ*, and *u*). Through correlating the parameters with ischemic outcomes (neurological deficit scores and infarct volumes), we expected that this model could serve as a promising approach to evaluate the disease outcomes.

## 2. Materials and Methods

### 2.1. Structure of Mini Network

**Figure 1:**
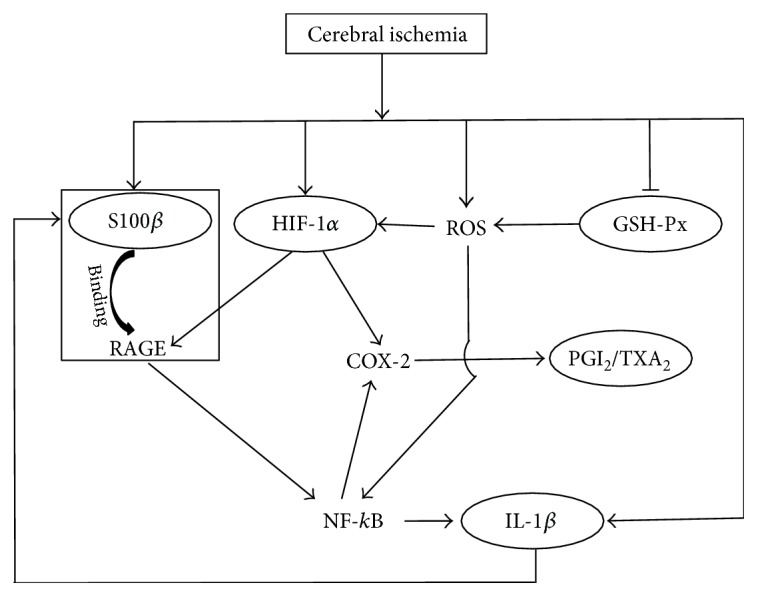


The structure of mini network is shown in Figure 1. The mini network was established based on the interactions among the related biomarkers.

### 2.2. Chemicals and Reagents

Chemicals and solvents were of analytical grade. Edaravone injections were supplied by Nanjing Yewin Pharmaceutical Co. Ltd. (Nanjing, China). ELISA kits were purchased from Wuhan Biological Engineering Co. Ltd. (Wuhan, China). RIA diagnostic kits were supplied by Beijing North Institute of Biological Technology. The commercial reagent kits for detection of GSH-Px activities were provided by Nanjing Jiancheng Bioengineering Institute (Nanjing, China). Monofilament nylon sutures were purchased from Beijing Shadong Biological Technology Co. Ltd. (Beijing, China). The multiplex kits were obtained from EMD Millipore Corporation (Millipore, Billerica, USA).

### 2.3. Animals and Experimental Model

Handling and experimental procedures on all animals were approved by the Animal Ethics Committee of China Pharmaceutical University. Male Sprague-Dawley rats weighing 250–280 g were obtained from SIPPR-BK Laboratory Animal Co. Ltd. (Shanghai, China). Rats were kept in a colony room under controlled temperature (25 ± 2°C) with a 12 : 12 light-dark cycle and provided with free access to water and a standard diet. Rats were fasted 8 hours prior to surgery. Rats underwent permanent middle cerebral artery occlusion surgery to produce a CI model [40]. Rats were divided into three groups: (1) the sham operation group (SHAM group, *n* = 6), (2) the permanent middle cerebral artery occlusion group (MCAO group, *n* = 6), and (3) the edaravone-treated group (EDA group, *n* = 6; 3 mg/kg body weight, i.p.). All the rats were anesthetized with chloral hydrate (0.3 g/kg, body weight, i.p.) and fixed in a supine position. The right common carotid artery (CCA), external carotid artery (ECA), and internal carotid artery (ICA) were carefully isolated through a midline cervical incision. A monofilament nylon suture (diameter: 0.26 mm; length: 40 ± 2 mm) with a rounded tip (diameter: 0.36 ± 0.02 mm) was introduced into the CCA lumen and gently advanced into the ICA until feeling a resistance. In sham-operated rats, the CCA, ECA, and ICA were exposed without inserting the filament into the internal carotid artery. EDA (3 mg/kg, i.p.) was administered at 5 minutes after MCAO surgery, once a day for a week. The SHAM and MCAO groups were treated with an equivalent volume of saline. The tail vein blood samples were collected in tubes containing EDTA-2K before and 1, 3, 5, and 7 days after cerebral ischemia. Neurological deficit evaluations were carried out by two observers who were blinded to the identities of the experimental groups using 3 tests.

#### 2.3.1. Postural Reflex Test [41]

Rats were suspended by their tails 33 cm above the floor and monitored for abnormal posture. Rats outstretching both forelimbs without other neurological deficits were recorded as score 0. A scale of 1 to 4 was established based on flexing the wrist mildly, flexing the wrist and elbow, adducting the contralateral shoulder, and rotating the contralateral limb.

#### 2.3.2. Push Resistance Test [41]

Rats were pushed to slide to the contralateral side with gentle pressure behind the left shoulder and then the right shoulder. Intact rats displaying distinctive symmetrical resistance in both directions achieved 0. Rats, exhibiting a declined resistance to the lateral push, scored 1 to 3 based on the degrees of decline in resistance.

#### 2.3.3. Bilateral Forepaw Grasp [42, 43]

Two-forelimb strength was detected. Rats were lifted to make the forelimbs touch a 2 mm diameter steel rod. Rats displaying the normal grasp behavior in the two forelimbs scored 0. Really unable to grasp with the forepaws, rats were graded 3. According to the above test results, the value is 0 to 10 points.

The higher scores the rat awarded, the more serious the behavior disorder was. Neurological deficit evaluations were performed before and 1, 3, 5, and 7 days after MCAO.

### 2.4. Detection of Multiple Biomarkers

Plasma HIF-1*α* and S100*β* levels were detected using ELISA kits (Cusabio Biotech Co. Ltd., Wuhan, China). All the procedures of measurements were performed according to the manufacturer's instruction in ELISA kits. PGI_2_ and TXA_2_ are very instable and easy to be converted to their inactive metabolites 6-keto-PGF_1*α*_ and TXB_2_, respectively. Therefore, we measured the levels of 6-keto-PGF_1*α*_ and TXB_2_. The detailed procedures of measurements followed the manufacturer's instruction in radioimmunoassay reagent kits (Beijing North Institute of Biological Technology, Beijing, China). Plasma IL-1*β* levels were measured using multiplex kits from Millipore (USA). The plasma GSH-Px activities were determined following the manufacturer's instruction in commercial reagent kits (Nanjing Jiancheng Bioengineering Institute, Nanjing, China).

### 2.5. The Development of a Mathematical Model

We introduced a mathematical model to integrate the information from the multiple biomarkers and integrally describe the disruption state of the mini network after cerebral ischemia. The disruption of the mini crosstalk network is mainly reflected by three disruption parameters (*k*, *φ*, and *u*). The parameter *k* responds to the magnitude of multiple biomarkers' change, *φ* describes the inconsistency of multiple-marker levels' variation, and *u* integrates the information of *k* and *φ* to describe the deviation between the disease and normal state. The three parameters *k*, *φ*, and *u* are calculated as follows [44]:

(1) $$\begin{array}{c} k=\frac{\left|V_{a}\right|}{\left|V_{b}\right|}, \end{array}$$

(2) $$\begin{array}{c} \varphi =\cos^{-1}\frac{V_{a}•V_{b}}{\left|V_{a}\right|\left|V_{b}\right|}, \end{array}$$

(3) $$\begin{array}{c} u=\sqrt{\left(V_{a}-V_{b}\right){\left(V_{a}-V_{b}\right)}^{T}}. \end{array}$$

In the network model, *V*_*a*_ is a vector for describing the disease state, which reflects the levels or activities of the related biomarkers of rats in the MCAO and EDA groups. *V*_*b*_ is a vector for describing the normal state, which reflects levels or activities of the related biomarkers in the SHAM group. A detailed description of the *V*_*a*_ and *V*_*b*_ is provided in the Supplementary Material available online at https://doi.org/10.1155/2017/9506527. *T* in (3) represents vector transposition.

### 2.6. Measurement of Infarct Volume

All animals were sacrificed 7 days after initiation of MCAO. The brains were removed rapidly from the skull, prepared into 2 mm thick coronal sections, and incubated with 2% 2,3,5-triphenyltetrazolium chloride (TTC; Sigma Chemical Co., St. Louis, MO, USA) for 30 min. These sections were fixed at 37°C by immersing in 10% formalin overnight. The normal tissues were stained red, while the infarcted tissues remained white. The fixed tissues were weighed and photographed by image analysis software. The total infarct area was determined by adding infarct areas of all sections and then multiplying by the thickness (2 mm) of the brain sections. The percent of infarct volume was determined by the following formulation [45]:

(4) $$\begin{array}{c} infarct\;volume\;percent=\frac{V_{infarcted\;hemisphere\;tissue}}{V_{control\;hemisphere}}\times 100\%. \end{array}$$

*V*_infarcted hemisphere tissue_ is the volume of the infarcted hemisphere tissue. *V*_control hemisphere_ is the volume of the control hemisphere.

### 2.7. Statistical Analysis

Data was expressed as means ± SEM. The one-way ANOVA was used for multiple comparisons followed by Tukeys' post hoc test. A value of *p* < 0.05 was considered statistically significant. Correlation analysis was analyzed with SPSS 19.0.

## 3. Results

### 3.1. Longitudinal Studies of the Multiple Markers

**Figure 2:**
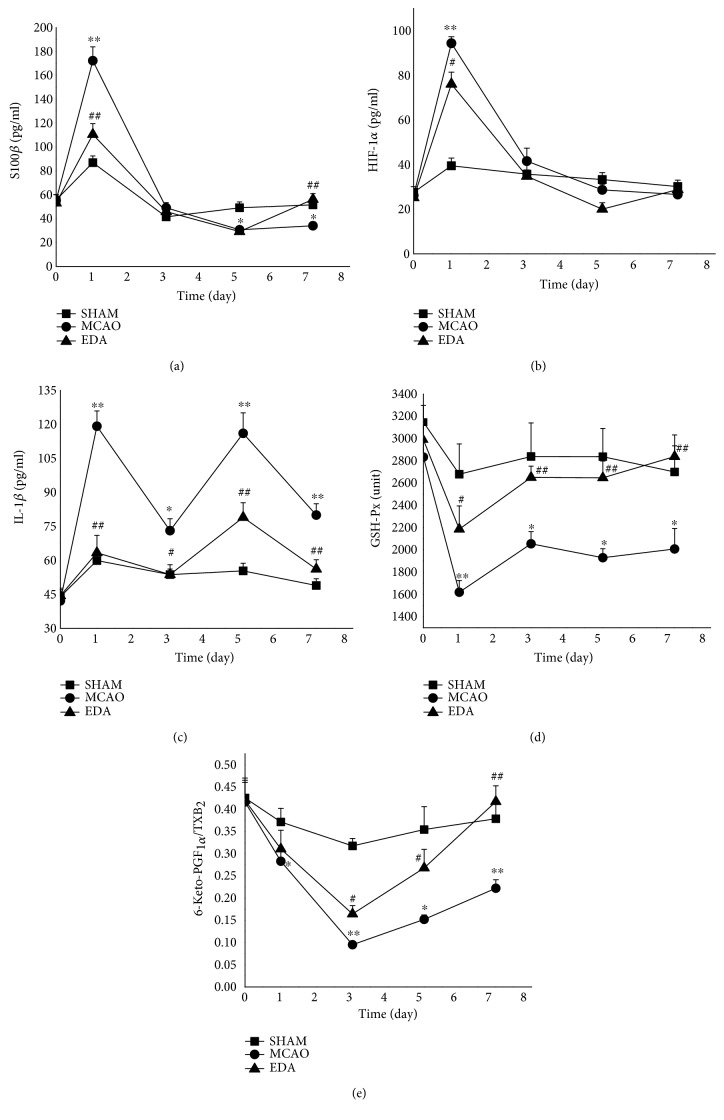


To develop a mathematical model of multibiomarker in the crosstalk network, the levels or activities of the related biomarkers were measured before and 1, 3, 5, and 7 days after MCAO (Figure 2). After the first day posttreatment with MCAO, the plasma S100*β* and HIF-1*α* levels significantly increased, while they were markedly reduced by EDA (*p* < 0.01) (Figures 2(a) and 2(b)). The concentrations of plasma IL-1*β* showed a fluctuant patterns with two peaks on both the first day and fifth day in the MCAO group (Figure 2(c)). IL-1*β* levels markedly declined after the treatment with EDA on the first day. The activities of GSH-Px decreased rapidly on the first day and then recovered gradually in the MCAO group (Figure 2(d)). A notable enhancement of GSH-Px activities was observed in the EDA group. The ratio of 6-keto-PGF_1*α*_ and TXB_2_ signally reduced 3 days after MCAO onset, and EDA significantly increased the ratio (Figure 2(e)).

### 3.2. Crosstalk Network Balance Maps and Parameters

**Figure 3:**
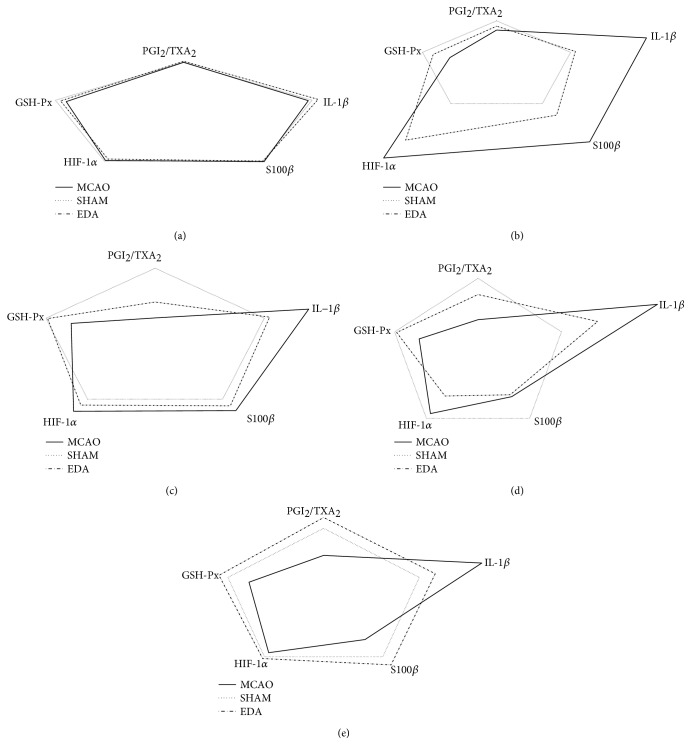


**Figure 4:**
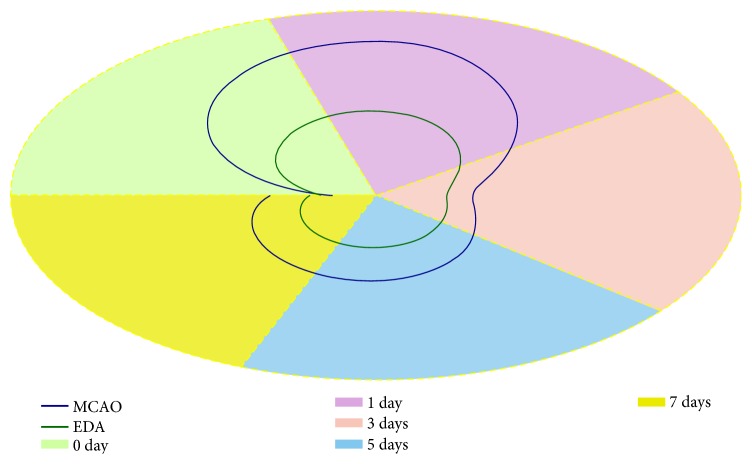


The mini crosstalk network balance maps of the related biomarkers at different time points were displayed (Figure 3). To further determine the extent of the mini network disruption, three mini network disruption parameters (*k*, *φ*, and *u*) were calculated (Table 1 and Figure 4). The parameter *u* value of the MCAO group maximized on the first day and was decreased by EDA after CI. In summary, these results showed that the most serious disruption of the mini network emerged on the first day, but EDA could markedly alleviate the state of disruption.

### 3.3. Neurological Deficit Score

**Figure 5:**
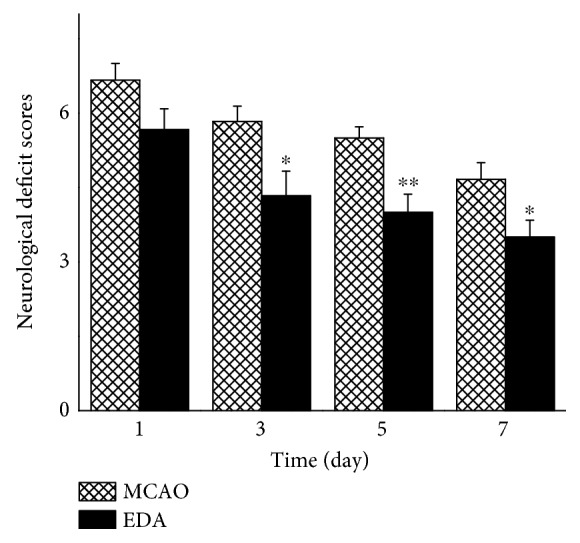


Neurological assessment indicated that all the preoperative animals scored 0. The neurological functions of sham-operated animals were not affected by anesthesia or surgical procedure and maintained 0 score until 7 days. The neurological scores significantly decreased in the MCAO group (Figure 5). Compared with those of the MCAO group, the neurological deficit scores markedly decreased in the EDA group from 3 to 7 days.

### 3.4. Cerebral Infarct Volume

**Figure 6:**
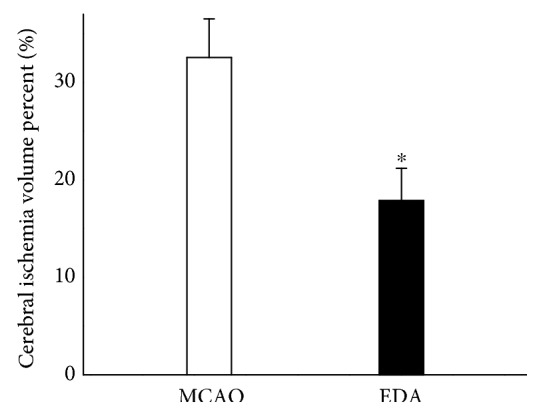


Infarct volume of all the rats except for the sham-operated animals on the 7th day is presented in Figure 6. EDA remarkably reduced infarct volume after MCAO.

### 3.5. Correlations between the Mini Network Disruption Parameter (*u*) Value and the Outcomes of Cerebral Ischemia (Neurological Deficit Score and Cerebral Infarct Volume)

The results showed that *u* values correlated significantly with neurological deficit scores (*R*^2^ = 0.727, *p* < 0.01). Moreover, there was a significant correlation between *u* values and cerebral infarct volumes (*R*^2^ = 0.799, *p* < 0.01) on the 7th day.

### 3.6. Correlation between the Levels of HIF-1*α* and S100*β*

There was a significant correlation between the levels of HIF-1*α* and S100*β* (*R*^2^ = 0.871, *p* < 0.01).

## 4. Discussion

To develop a mathematical model of a mini crosstalk network, the levels or activities of the related plasmatic biomarkers in rats were measured before and 1, 3, 5, and 7 days after ischemia. All the related biomarkers changed significantly after CI onset, which was in line with the previous reports. Moreover, the results verified that the physiopathology pathways, including oxidative stress, inflammation, neuronal damage, and hypoxia-inducible factor, were affected by CI. Interestingly, our results showed that there was a significant correlation between HIF-1*α* and S100*β* levels (*R*^2^ = 0.871, *p* < 0.01) in plasma. It was possible that the dramatic elevation of HIF-1*α* levels could promote the overexpression of the downstream targets after ischemia, such as VEGF, MMP-2, and MMP-9 [13, 46]. Then, these downstream targets can increase the blood brain barrier (BBB) permeability [47–49]. Due to the dysfunction of BBB, S100*β* was released or secreted by an activated astrocyte following CI to leak into the peripheral blood [50, 51]. However, the exact cellular and molecular mechanisms should be demonstrated using the related experiments in the future. In this study, no other significant correlations were found between the different plasmatic biomarkers. It was possible that the time courses of these biomarkers were pretty inconsistent except for S100*β* and HIF-1*α*.

Based on the significant changes of these related biomarkers, we suggested that the comprehensive information from these biomarkers might be more theoretically leveraged for the disease outcome evaluation. Therefore, we developed a mathematical model to integrate the information from these biomarkers for evaluating the disease outcome. By developing the mathematical model, we achieved three disruption parameters which could quantitatively describe the disruption state of the mini network after CI. The parameter *k* responds to alterations of all these biomarker levels or activities. The smaller the change of these biomarker levels or activities gets, the closer the *k* value will be. However, if the changed magnitudes of the biomarker levels are similar but the changed directions are opposite, all the *k* could remain the same value. In other words, *k* value could not fully reflect the changes of these biomarker levels or activities in plasma. The parameter *φ* is a response to the inconsistency of the variation of the multiple-biomarker levels or activities in plasma. However, *φ* value does not describe the magnitudes of alteration of the biomarker levels or activities. Therefore, no stand-alone parameter (*k* or *φ*) value could fully reflect the alterations of these biomarker levels or activities in plasma. The results of correlation analysis also show that the value of *k* or *φ* is not obviously correlated with the disease outcome. Therefore, *k* and *φ* values could not be used to evaluate the disease outcome but reflect a specific feature of the alterations of these biomarker levels or activities. Then, the parameter *u* was introduced to integrate the information provided by *k* and *φ.* In the current study, *u* values showed significant change in the MCAO group on the first day. It implied that there was a severe disruption of the mini network after CI. Under the intervention of EDA, the disruption of the mini network was rapidly improved during the subacute phase of CI. The trajectory charts clearly displayed this trend. The neurological deficit scores showed the similar trend. Therefore, bivariate correlation analysis was used to examine the correlation between *u* values and neurological deficit scores and/or infarct volumes. The results showed that the parameter *u* values significantly correlated with the cerebral ischemic outcomes. Our current study suggests that *u* has an important reference value for assessing the disease outcome. The balance maps may intuitively display the mini network disruption of cerebral ischemic rats. The integral dynamic parameter *u* may serve as a promising indictor for evaluating the disease outcome of CI. Therefore, the mathematical model may offer a new way for evaluating the disease outcome of cerebral ischemic rats.

## Supplementary Material

The definition of Va and Vb

## Figures and Tables

**Table 1:** The values of the two integral disruption parameters (*k* and *φ*) before and 1, 3, 5, and 7 days after MCAO.

Group	Time (day)					
		0	1	3	5	7
MCAO	*k*	0.965 ± 0.039	1.946 ± 0.109	1.109 ± 0.077	1.283 ± 0.118	1.036 ± 0.062
	*φ*	0.161 ± 0.029	0.349 ± 0.023	0.342 ± 0.051	0.543 ± 0.044	0.444 ± 0.043
EDA	*k*	0.972 ± 0.044	1.393 ± 0.074^∗∗^	0.967 ± 0.096	0.988 ± 0.088	1.066 ± 0.039
	*φ*	0.209 ± 0.025	0.362 ± 0.032	0.241 ± 0.047	0.386 ± 0.033^∗^	0.251 ± 0.036^∗∗^

Data was expressed as means ± SEM. ^∗^*p* < 0.05, ^∗∗^*p* < 0.01 compared with the MCAO group.
