# Supplementary material for: A Promising Approach to Integrally Evaluate the Disease Outcome of Cerebral Ischemic Rats Based on Multiple-Biomarker Crosstalk
Source: Dis Markers. 2017 May 25;2017:9506527. doi: 10.1155/2017/9506527 (PMC5463200; doi:10.1155/2017/9506527)
Supplement: Supplementary file 2 [file 9506527.f2.docx]

Supplementary Material:

The disruption of the mini network is reflected by matrixs and vectors. The activities or levels of different biomarkers at different time points are defined as $c_{i}^{b}$, which denotes the activity or concentration of ith marker at the bth time point. The different markers have different orders of magnitude，which could induce the computational error. To avoid the computational error, the ratio of $\frac{c_{i}^{b}}{c_{i}^{l}}(r_{i}^{b})$, is used to replace $c_{i}^{b}$.

$$r_{i}^{b}=\frac{c_{i}^{b}}{c_{i}^{1}}$$

Where $c_{i}^{l}$is defined as the concentration or activity of the ith marker at the first time point before middle cerebral artery occlusion and is considered to be the normal state. A matrix is established to describe the state of the biomarkers at different time points.

$$R_{n\times m}=\left(\begin{matrix} 1 & r_{1}^{2} & \cdots& r_{1}^{m} \\ 1 & r_{2}^{2} & \ldots& r_{2}^{m} \\ \vdots& \vdots& \ddots& \vdots\\ 1 & r_{n}^{2} & \cdots& r_{n}^{m} \end{matrix}\right)$$

Vectors are used to describe the disruption at different time points. The bth column vector in *R_n × m_* is extracted to form a new vector, describing the state of the module at the bth time point.

$$V_{a}=\left(r_{1}^{1},r_{2}^{1},r_{3}^{1}\cdots r_{n}^{1}\right)$$

$$V_{b}=\left(r_{1}^{b},r_{2}^{b},r_{3}^{b}\cdots r_{n}^{b}\right)b=2,3\cdots m$$

Va is a vector for describing the disease state, which reflects the levels or activities of the related biomarkers of rats in MCAO and EDA groups. *V_b_* is a vector for describing the normal state, which reflects levels or activities of the related biomarkers in SHAM group.
